# Phase shift optimization in reconfigurable intelligent surface-assisted UAV in hierarchical aerial computing networks

**DOI:** 10.1038/s41598-026-38514-7

**Published:** 2026-03-03

**Authors:** Basma Diaa, Ibrahim I. Ibrahim, Ahmed M. Abdelhaleem, Mostafa M. Abdelhakam

**Affiliations:** Department of Electronics and Communications Engineering, Faculty of Engineering, Helwan University, Cairo, Egypt

**Keywords:** Aerial computing, unmanned aerial vehicle (UAV), High altitude platform (HAP), Reconfigurable intelligent surfaces (RIS), Mobile edge computing (MEC), Resource allocation, Matching game theory, Riemannian conjugate gradient (RCG), Engineering, Mathematics and computing

## Abstract

The evolution toward 6G wireless networks necessitates innovative solutions to support the massive Internet of Things (IoT) deployments with unprecedented computational and communication requirements, motivating this A comprehensive framework that integrates Reconfigurable Intelligent Surface (RIS) technology with hierarchical aerial computing networks by combining RIS-equipped Unmanned Aerial Vehicles (UAVs) operating as mobile edge computing nodes with High-Altitude Platforms (HAPs) to create a three-tier computing hierarchy addressing the limitations of conventional terrestrial infrastructure. The system model encompasses RIS-equipped UAVs serving terrestrial IoT devices with a single HAP providing high-capacity computational resources, where the RIS phase optimization is formulated as a Riemannian conjugate gradient problem on complex circle manifolds to maximize total system throughput while naturally handling unit modulus constraints through a three-stage sequential decomposition approach. Extensive Monte Carlo simulations demonstrate significant performance improvements over the comparable algorithm without RIS enhancement, with the RIS-enhanced system achieving 18% throughput improvement, near-linear scalability serving approximately 100% of available IoT devices compared to the algorithm In the comparable algorithm at 100 devices, a 95% task completion rate was maintained across all network loads versus 79–80% for the algorithm compared to the comparable algorithm. The results validate the potential of RIS-enabled aerial networks as a transformative solution for scalable and efficient 6G IoT services, with enhanced channel quality from intelligent phase configuration, enabling superior resource utilization and service provisioning in hierarchical computing architectures, establishing key contributions including novel RIS-aerial computing integration, advanced Riemannian manifold optimization with superior convergence properties, unified resource allocation combining stable matching theory with externality elimination, comprehensive performance analysis demonstrating practical viability, and real-world implementation considerations for future multi-UAV scenarios and energy-efficient designs.

## Introduction

The evolution toward 6G wireless networks necessitates innovative solutions to support massive IoT deployments with stringent quality of service requirements^1,2^.Recent advances in Reconfigurable Intelligent Surfaces (RIS) offer unprecedented opportunities to enhance wireless links through intelligent signal manipulation^3,4^. While hierarchical aerial computing networks using HAP and UAV have shown promise for providing mobile edge computing services^5^, their communication performance remains fundamentally limited by adverse channel conditions and interference, particularly in dynamic three-dimensional service topologies. However, integrating RIS with multi-tier aerial computing platforms presents unique and largely unaddressed challenges. These challenges stem from the complex coupling between RIS phase configuration, dynamic resource allocation, and the hierarchical offloading decisions across IoT, UAV, and HAP tiers.

Existing literature has made significant progress in two distinct domains. On one hand, works like^5^ have developed sophisticated resource allocation and matching algorithms for HAP-UAV-IoT hierarchies, but without considering RIS for channel enhancement. On the other hand, RIS optimization has been extensively studied for simple, static, or single-tier communication scenarios^4,6^. A critical research gap exists in jointly optimizing the RIS phase configuration within a dynamic, three-tier (IoT-UAV-HAP) computation offloading framework. Prior approaches typically treat RIS beamforming and hierarchical task scheduling as separate problems. This decoupling fails to capture the essential system dynamics: the optimal RIS phase shifts for IoT-UAV links directly influence the uplink data rate, which in turn determines the latency and feasibility of local UAV computation versus offloading to the HAP. Consequently, there is a lack of a unified optimization framework that: (1) natively and efficiently handles the unit modulus constraints of passive RIS elements, (2) coordinates stable device-association in the presence of resource competition and preference interdependencies, and (3) jointly makes intelligent computation placement decisions across the aerial hierarchy based on RIS-enhanced channel states. This paper bridges this gap by introducing a novel RIS-assisted hierarchical t computing system. We address the joint optimization of RIS phase configuration and multi-tier resource allocation in an integrated model. The primary challenge lies in maximizing the total system throughput while ensuring stable matching, respecting individual energy budgets, and guaranteeing stringent end-to-end delay constraints for all tasks. To this end, we develop a hybrid algorithmic solution that decomposes the complex problem into manageable yet coordinated sub-problems.

### Related work

Computation offloading has gained significant attention in recent years as a means to overcome the limited processing and energy resources of IoT devices. With the proliferation of IoT devices and the advent of 5G and beyond wireless technologies, traditional cloud computing approaches face significant challenges in meeting the stringent latency and bandwidth requirements of emerging applications. Aerial platforms, particularly UAVs and HAPs, have emerged as promising enablers for MEC due to their flexibility, wide coverage, and ability to complement terrestrial infrastructures. For example in^7^ proposed UAV-assisted MEC architectures where UAVs act as relays forwarding computation tasks from IoT devices to ground edge servers. The authors formulated a joint optimization problem involving IoT-UAV-ES associations and UAV deployment topology, developing a Restricted Three-Sided Matching with Size and Cyclic Preference (R-TMSC) algorithm. Hierarchical aerial computing architectures were further explored in^5^, Jia investigated joint trajectory and resource optimization in UAV-HAP MEC networks assisted by RIS, demonstrating significant throughput gains through tiered offloading. Similarly, Zhao et al.^8^ examined energy-latency trade-offs in RIS-assisted aerial computing, highlighting the role of intelligent surfaces in improving energy efficiency. Li et al.^9^ further expanded the scope to multi-HAP multi-UAV edge computing networks, incorporating mobility-aware dynamic resource allocation.

For Computation Offloading Methodologies^10^, cover fundamental elements of DRL algorithm design and highlighting superior perception and decision-making capabilities compared to traditional optimization. The integration of blockchain with MEC is explored in^11^ which presents an A3C-AHP framework for multi-user optimization in blockchain-enabled IoT systems. For industrial IoT applications^12^, addresses computation offloading in multi-server edge computing environments with joint load balancing and security considerations, incorporating fuzzy security mechanisms.

A comprehensive study on resource provisioning and optimization in edge computing is provided in^13^, which offers a holistic review of static, dynamic, and user-centric resource allocation models and examines the integration of Software Defined Networks (SDN) for enhancing resource orchestration. The survey highlights critical challenges, including scalability, interoperability, and security in managing dynamic and heterogeneous infrastructures.

RIS technology has emerged as a transformative solution for improving wireless communication by creating controllable electromagnetic environments^14,15^. Recent advancements have focused on RIS integration with aerial platforms with studies exploring intelligent reflecting surfaces for multi-UAV NOMA communications and implementation using compressive sensing and deep learning techniques^16^. Huang et al.^17^ provide a comprehensive survey on RIS-assisted UAV communications, covering joint beamforming, trajectory design, and energy efficiency. Nguyen^18^ investigate joint UAV trajectory and RIS phase shift optimization for IoT data collection, while Wang^19^ apply deep reinforcement learning for secure RIS-assisted UAV communications in dynamic environments. In the context of phase optimization, Mei^20^ advance Riemannian optimization techniques for RIS phase design in multi-user MIMO systems, offering methodological enhancements relevant to manifold-based approaches. Practical implementation aspects are addressed by Zhou^21^, who provide experimental validation of RIS-assisted UAV links in urban scenarios.

### Contribution

The key contributions of this study are outlined in the following points:

- We propose the first comprehensive framework that integrates RIS technology with hierarchical aerial computing networks, combining RIS-equipped UAVs operating as mobile edge computing nodes with HAPs to create a three-tier computing hierarchy. Unlike existing works that consider either aerial computing or RIS enhancement separately, our integrated approach addresses both communication and computation challenges simultaneously, creating synergistic performance improvements beyond what either technology achieves independently.
- The RIS phase optimization is formulated as a Riemannian conjugate gradient problem on the complex circle manifolds, representing a significant methodological advancement over conventional optimization approaches. This geometric formulation naturally handles unit modulus constraints inherent in passive RIS elements while achieving superior convergence properties compared to penalty-based or alternating optimization methods commonly used in prior work. The manifold-based approach exploits the intrinsic geometric structure of the constraint space, enabling more efficient and stable optimization.
- We develop a three-stage sequential decomposition algorithm that uniquely combines (i) stable matching theory with externality elimination for IoT-UAV associations, addressing preference interdependencies that existing matching approaches ignore; (ii) Riemannian manifold optimization for RIS phase configuration; and (iii) intelligent heuristics for hierarchical UAV-HAP task distribution. Unlike prior hierarchical computing works that separate resource allocation from communication enhancement, our unified approach ensures that improved channel conditions from RIS optimization directly inform and enhance resource allocation decisions.
- We provide extensive simulation results demonstrating substantial improvements over comparable hierarchical aerial computing systems: 18% throughput improvement, near-linear scalability serving approximately 100% of available IoT devices (compared to plateauing at $\sim$80% in baseline approaches), 95% task completion rate maintained across all network loads versus 79–80% for conventional systems, and 28.6% reduction in average task processing delay. These results establish the practical viability and quantifiable benefits of RIS-enhanced aerial computing.
- Unlike theoretical studies that assume idealized conditions, we address real-world constraints including finite energy budgets, strict delay requirements, practical RIS hardware limitations (amplitude losses, phase resolution), and computational complexity considerations. We also analyze the energy-performance tradeoffs inherent in RIS operation and provide insights for system designers on when and how to deploy RIS-enhanced aerial computing platforms.

The combination of these contributions establishes a new paradigm for scalable and efficient 6G IoT services, where intelligent manipulation of the wireless environment through RIS technology fundamentally enhances the capabilities of hierarchical aerial computing architectures.

The rest of this work is arranged as follows. A comprehensive system model is presented in Section II, including the hierarchical network architecture, RIS-enhanced channel models, time and energy cost models, and the mathematical problem formulation. The algorithms are proposed in Section III, encompassing the stable matching algorithm with externality elimination for IoT-UAV associations, the Riemannian conjugate gradient algorithm for RIS phase optimization and the heuristic approach for UAV-HAP task offloading. Section IV provides extensive simulation results and performance evaluation, comparing the proposed RIS-enhanced system against baseline approaches across various metrics, including total computed data, energy consumption, and task completion rates. conclusions are offered in Section V. Finally, Section VI concludes the papers and outlines potential research directions for RIS-enhanced aerial computing networks. Besides, for clarity, the notations used in this work are listed in Table 1.

**Fig. 1:**
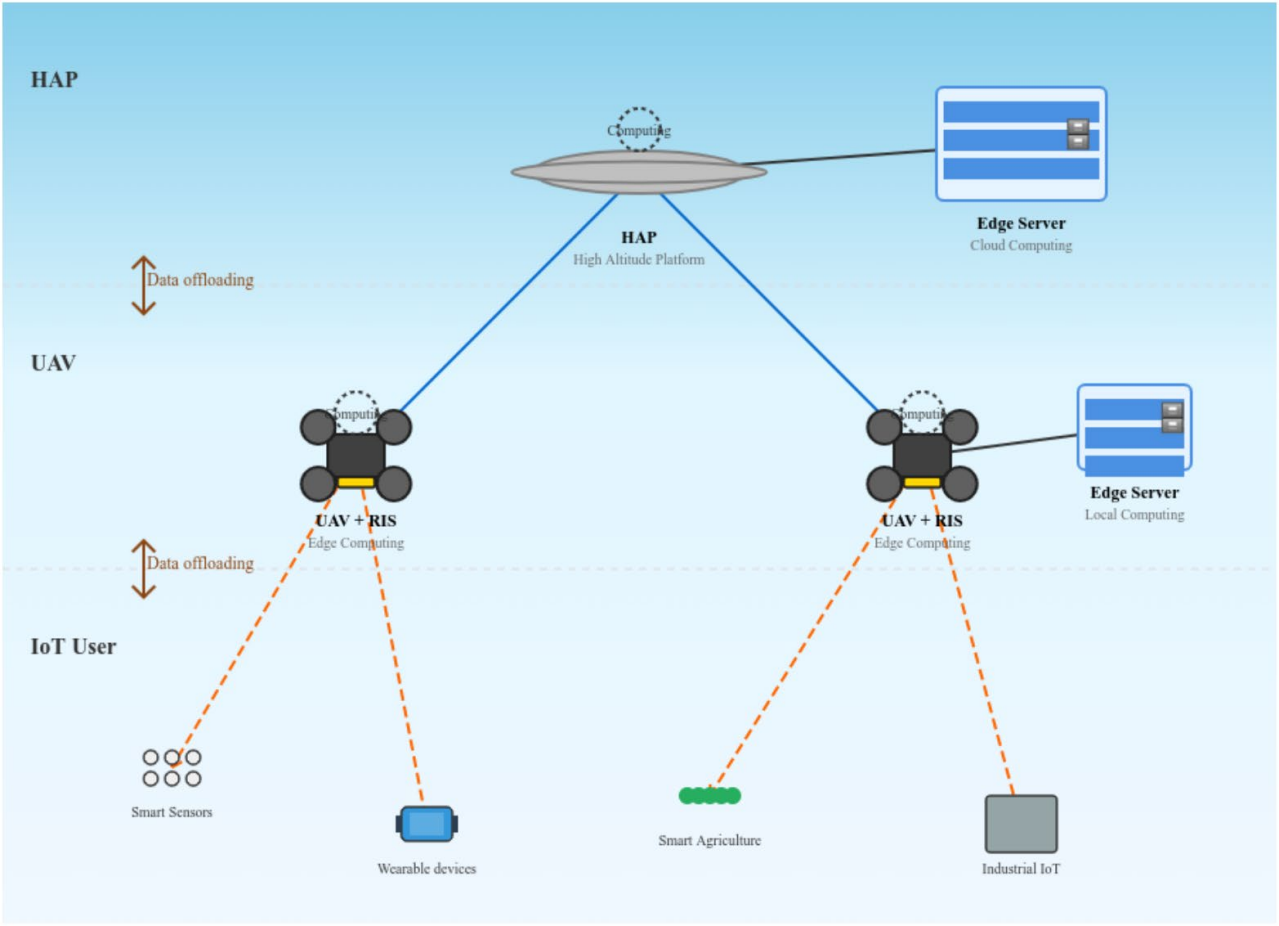
RIS-enhanced hierarchical aerial computing network

## System model

### Network architecture

As illustrated in Fig. 1, the hierarchical aerial computing framework is composed of an RIS-Assisted UAV and a single HAP in the air, and terrestrial IoT users in various applications, e.g., Smart Sensors, Wearable devices, Security Camera, Smart Agriculture, and Industrial IoT. We consider an uplink transmission scenario in an integrated terrestrial and non-terrestrial wireless network. The HAPS is located in the center of the coverage area at an altitude of 20 km^22^. Additionally, each UAV is equipped with a reconfigurable intelligent surface (RIS) to improve communication performance and improve data transmission rates between IoT devices and aerial platforms. In this work, we assume the RIS is integrated into the UAV platform (co-located). Both UAVs and HAPS are equipped with edge servers, and HAPS have a stronger load capacity than UAVs. Ground IoT users have various computing demands, but with limited computing capability, especially for small-sized IoT devices. As for the lightweight computation demands, IoT devices can complete computing locally. However, due to the limited computing and energy resources of IoT devices, the computation-intensive demands may not be completed locally by the IoT devices, and UAVs equipped with edge servers can provide the computing service for these IoT devices via data offloading. Furthermore, the payload for the computation of the UAV is limited and the computing tasks on the UAV may fail. In this case, HAPS with a stronger payload can assist UAVs in accomplishing the computation task from IoT devices. In such a way, the UAV serves as a relay for the data from IoT offloading to the HAPS, only performing binary computation offloading is considered in this model, i.e., the computing task has two choices: offloading to a UAV and computing by the edge server of the UAV, or offloading to the HAPS and computed by the edge server of the HAPS, according to the resource provision, as depicted in Fig. 1.

### Channel model

Each UAV is equipped with a Reconfigurable Intelligent Surface consisting of N passive reflecting elements. The RIS is strategically positioned on the UAV platform to optimize signal reflection between IoT devices and the UAV’s communication systems.

#### IoT to RIS-UAV link

The channel gain between IoT device *i* and RIS-UAV *u* is^4^:

1 $$\begin{aligned} G_{i,u}^{\text {RIS}}= G_{i,u}\cdot G^{\text {RIS}} \end{aligned}$$

where $G_{i,u}=\frac{G_0}{\Vert \textbf{q}_u - \textbf{q}_i\Vert ^2 + H_u^2}$ the line-of-sight channel gain between IoT i and UAV u (I2U), $G_0$ is the reference channel gain, $\textbf{q}_u$ and $\textbf{q}_i$ are the horizontal positions of UAV u and IoT i, respectively, $H_u$ is the UAV altitude, and $G^{\text {RIS}}$ is the RIS gain, which is determined by^23^

2 $$\begin{aligned} G^{\text {RIS}} = G_{\text {orient}}(\theta _u) \cdot \left| \sum _{n=1}^{N} \alpha _n(\phi _n) e^{j\varphi _n}\right| ^2 \end{aligned}$$

where $G_\text {orient}(\theta _u)$ is the orientation gain factor, $\alpha _n(\phi _n)$ is a phase-dependent attenuation reflection coefficient, *N* is the reflection elements, and $\varphi _n$ is the phase shift of N elements. The RIS-enhanced data rate is given by^6,24^:

3 $$\begin{aligned} R_{i,u}= BW_{iu} \cdot \log _2\left( 1 + \frac{P_i^{\text {tr}} G_{i,u}^{\text {RIS}} }{\sigma ^2}\right) \end{aligned}$$

where $BW_{iu}$ is the bandwidth of I2U channel, $P_i^{\text {tr}}$ is the transmitted power to transmit the data of IoT i to UAV u, and ${\sigma ^2}$ is the noise power density.

#### RIS-UAV to HAP link

The backhaul link from RIS-UAV *u* to HAP (U2H) follows free space propagation:

4 $$\begin{aligned} R_u = BW_u \cdot \log _2\left( 1 + \frac{P_u^{\text {tr}} G_u L_s L_l \cdot G^{\text {RIS}}}{k_B T_s BW_u}\right) \end{aligned}$$

where $BW_u$ is the bandwidth of U2H channel, $P_i^{\text {tr}}$ is the transmitted power to transmit the data of UAV u to HAPS, $G_u$ is the anntena power gain , $L_s = \left( \frac{c}{4\pi d_u f_u}\right) ^2$ is the free space path loss , $k_B$ is the Boltzmann constant, and $T_s$ denotes the system noise temperature.

### Time cost model

The total time cost for completing an IoT task consists of transmission delays and computation processing times across the hierarchical aerial computing framework.

#### Time cost for transmission

**IoT to UAV:** The time required to transmit data from IoT device *i* to UAV *u* is given by:

5 $$\begin{aligned} T_{iu} = \frac{K_i A_u^i}{R_{i,u}} \end{aligned}$$

where $K_i$ represents the data size of IoT *i*, $A_u^i \in \{0, 1\}$ indicates whether the task of IoT $i \in \mathscr {I}$ is offloaded to UAV *u*, and $R_{i,u}$ denotes the achievable data rate between IoT *i* and UAV *u*.

**UAV to HAP:** When a UAV relays IoT data to a HAP for computation, the transmission time is calculated using:

6 $$\begin{aligned} T_{u} = \frac{K_i B^{i,u}}{R_u} \end{aligned}$$

where $B^{i,u} \in \{0, 1\}$ indicates whether the task from IoT *i* is forwarded to HAP by UAV *u*.

#### Time cost for computing

**UAV Computing Time:** The time for UAV *u* to complete the computation task for IoT *i* is expressed as:

7 $$\begin{aligned} T_u^i = \frac{K_i \alpha _u^i}{C_u/\rho _u} = \frac{K_i \alpha _u^i \rho _u}{C_u} \end{aligned}$$

where $\alpha _u^i \in \{0, 1\}$ indicates whether the task of IoT $i \in \mathscr {I}$ is computed by UAV *u*, $\rho _u$ represents the computational resource cost per bit, and $C_u$ denotes the computation capability of UAV *u*.

**HAP Computing Time:** Similarly, the computation time at the HAP is given by:

8 $$\begin{aligned} T^i = \frac{K_i \delta _h^i}{C_h/\mu _h} = \frac{K_i \delta _h^i \mu _h}{C_h} \end{aligned}$$

where $\delta ^i \in \{0, 1\}$ indicates whether the task of IoT $i \in \mathscr {I}$ is computed by HAP, $\mu _h$ is the computational resource cost per bit for the HAP, and $C_h$ represents the HAP’s computation capability. The total time cost for IoT device *i* is:

9 $$\begin{aligned} T_i^{\text {total}} = \sum _{u \in \mathscr {U}} \left[ \frac{K_i A_u^i}{R_{i,u}} + \frac{K_i \alpha _u^i \rho _u}{C_u} + \frac{K_i B_{i,u}}{R_u} \right] + \frac{K_i \delta _i \mu }{C} \end{aligned}$$

This encompasses all transmission and computation delays in the hierarchical system, including IoT-to-UAV transmission, UAV computation, UAV-to-HAP transmission, and HAP computation, ensuring that the end-to-end the delay constraint is satisfied for successful task completion.

### Energy cost model

The energy consumption in the hierarchical aerial computing framework consists of operational energy and task-specific energy costs for IoT devices, UAVs, and HAPs. Each entity has distinct energy consumption patterns based on its computational and communication activities.

#### Energy cost of IoT

The total energy cost $E_i^c$ of IoT device *i* comprises the basic operational energy and transmission energy for data offloading:

10 $$\begin{aligned} E_i^c = E_i^o + \sum _{u \in \mathscr {U}} \frac{P_i^{tr} K_i A_u^i}{R_{i,u}} \end{aligned}$$

where $E_i^o$ represents the basic operational energy cost of IoT *i*, $P_i^{tr}$ denotes the transmission power from IoT *i* to UAV *u*, $K_i$ is the data size, and $R_{i,u}$ represents the achievable data rate between IoT *i* and UAV *u*.

#### Energy cost of UAV

The total energy cost $E_u^c$ of UAV *u* includes basic operation, computation processing, and data transmission to HAPs:

11 $$\begin{aligned} E_u^c = E_u^o + \sum _{i \in \mathscr {I}} \varsigma _u C_u^2 K_i \rho _u \alpha _u^i + \sum _{i \in \mathscr {I}} \frac{P_u^{tr} K_i B^{i,u}}{R_u} + E_u^{RIS} \end{aligned}$$

where $E_u^o$ is the basic operational energy cost (e.g., hovering energy), $\varsigma _u$ represents the energy consumption coefficient depending on the UAV’s processor chip architecture, $C_u$ is the computing capability of UAV, $\rho _u$ is the computational resource cost per bit, $P_u^{tr}$ is the transmission power from UAV to HAP, $B^{i,u}$ represents the data forwarding decision, $R_u$ is the UAV-to-HAP data rate, and $E_u^{RIS}$ account for additional energy costs related to reconfigurable intelligent surfaces or other advanced technologies.

#### Energy cost of HAP

The total energy cost $E^c$ of the HAP system primarily consists of basic operational energy and computation energy:

12 $$\begin{aligned} E^c = E^o + \sum _{i \in \mathscr {I}} \varsigma C^2 K_i \delta _i \mu \end{aligned}$$

where $E^o$ represents the basic operational energy cost of the HAP, $\varsigma$ is the energy consumption coefficient for the HAP’s processor, *C* is the computing capability of HAP, and $\mu$ represents the computation resource cost of HAP. These energy models ensure that the hierarchical aerial computing system operates within the energy budget constraints of each platform while maximizing the overall system performance.

#### RIS energy cost

The energy consumption of the Reconfigurable Intelligent Surface mounted on each UAV, denoted as $E_{u}^{\text {RIS}}$, is modelled as the total energy required to operate the RIS over the UAV’s active mission duration. This accounts for both the power dissipated by the individual reflecting elements and the control circuitry responsible for configuring their phase shifts.

For a passive RIS comprising $N$ tunable reflecting elements, the total steady-state power consumption $P_{u}^{\text {RIS}}$ is given by^25,26^:

13 $$\begin{aligned} P_{u}^{\text {RIS}} = N \cdot P_{\text {element}} + P_{\text {ctrl}} \end{aligned}$$

where $P_{\text {element}}$ is the bias power required per reflecting element, $P_{\text {ctrl}}$ is the power consumption of the RIS control unit that computes and sets the phase configuration $\phi$.

The total energy consumed by the RIS over an operational time window $T_{\text {op}}$ is therefore:

14 $$\begin{aligned} E_{u}^{\text {RIS}} = P_{u}^{\text {RIS}} \cdot T_{\text {op}} \end{aligned}$$

Where $T_{{op}}$ represents a UAV’s active computing/communication mission phase.

### Problem formulation

The objective is to maximize the total volume of IoT data successfully processed by the hierarchical aerial computing platforms (RIS-equipped UAVs and HAP) and enhance communication performance by implementing RIS phase optimization subproblem^27^ which is formulated as:

15 $$\begin{aligned} \text {(P-RIS):} \quad \max _{\phi } R_{i,u}(\phi ) \end{aligned}$$

The formulated optimization problem (P0) represents a mixed-integer nonlinear programming (MINLP) problem that exhibits strong coupling between discrete resource allocation decisions and continuous RIS phase configuration variables.

16 $$\begin{aligned} (P0): \quad \max _{A, \alpha , B, \delta ,\phi } \sum _{i \in \mathscr {I}} \sum _{u \in \mathscr {U}} K_i \left( \alpha _u^i + \delta ^i\right) \end{aligned}$$

16.a $$\begin{aligned}&\text {s.t.} \quad \sum _{u \in \mathscr {U}} A_u^i \le 1, \forall i \in \mathscr {I}, \end{aligned}$$

16.b $$\begin{aligned}&\quad \alpha _u^i + B^{i,u} = A_u^i, \forall i \in \mathscr {I}, u \in \mathscr {U}, \end{aligned}$$

16.c $$\begin{aligned}&\quad \sum _{i \in \mathscr {I}} A_u^i \le N_u, \forall u \in \mathscr {U}, \end{aligned}$$

16.d $$\begin{aligned}&\quad \delta ^i \le \sum _{u \in \mathscr {U}} B^{i,u}, \forall i \in \mathscr {I}, \end{aligned}$$

16.e $$\begin{aligned}&\quad \sum _{i \in I} \alpha _i^u \rho _u \le C_u, \quad \forall u \in U, \end{aligned}$$

16.f $$\begin{aligned}&\quad \sum _{i \in I} \delta _i \mu \le C, \end{aligned}$$

16.g $$\begin{aligned}&\quad E_i^c \le E_i, \forall i \in \mathscr {I}, \end{aligned}$$

16.h $$\begin{aligned}&\quad E_u^c \le E_u, \forall u \in \mathscr {U}, \end{aligned}$$

16.i $$\begin{aligned}&\quad E^c \le E, \end{aligned}$$

16.j $$\begin{aligned}&\quad T_i^{total} \le D_i, \forall i \in \mathscr {I}, \end{aligned}$$

16.k $$\begin{aligned}&\quad \sum _{n=1}^{N} |\alpha _n|^2 \le N, \end{aligned}$$

16.l $$\begin{aligned}&\quad \phi _n \in [0, 2\pi ], \forall n \in \{1, 2, \ldots , N\}, \end{aligned}$$

16.m $$\begin{aligned}&\quad 0 \le \alpha _n \le 1, \forall n \in \{1, 2, \ldots , N\}, \end{aligned}$$

16.n $$\begin{aligned}&\quad |e^{j\phi _n}| = 1, \forall n \in \{1, 2, \ldots , N\}. \end{aligned}$$

16.o $$\begin{aligned}&\quad A_u^i, \alpha _u^i, B^{i,u} \in \{0,1\}, \forall i \in \mathscr {I}, u \in \mathscr {U}, \end{aligned}$$

16.p $$\begin{aligned}&\quad \delta ^i \in \{0,1\}, \forall i \in \mathscr {I} \end{aligned}$$

16.q $$\sum\limits_{{i \in {\mathcal{I}}}} {K_{i} } \le \min \left\{ {\sum\limits_{{u \in {\mathcal{U}}}} {\frac{{C_{u} }}{{\rho _{u} }}} + \frac{C}{\mu },\quad \sum\limits_{{i \in {\mathcal{I}}}} {K_{i} } \cdot \mathbb{1} (E_{i}^{{{\mathrm{tr}}}} \le E_{i} )} \right\}.$$

where we have $A = \{A_u^i, \forall i \in \mathscr {I}, u \in \mathscr {U}\}$, $\alpha = \{\alpha _u^i, \forall i \in \mathscr {I}, u \in \mathscr {U}\}$, $B = \{B^{i,u}, \forall i \in \mathscr {I}, u \in \mathscr {U}\}$, $\delta = \{\delta ^i, \forall i \in \mathscr {I}\}$, and $\phi = \{\phi _n, \forall n \in \{1, 2, \ldots , N\}\}$, denoting the variable vectors of IoT data offloading to the UAV, UAV-based MEC, IoT data offloading to the HAP, HAP-based MEC, and RIS phase configuration, respectively. The constraints of problem P0 can be categorized into five groups based on their functional roles in the hierarchical aerial computing system:

*Association and Flow Control Constraints:* Constraints **(16.a)**-**(16.d)** establish the fundamental communication and data flow rules. The IoT association constraint **(16.a)** prevents communication conflicts by limiting each IoT device to connect with at most one UAV, which is essential for RIS beamforming effectiveness. The flow conservation constraint **(16.b)** implies the data flow conservation at a UAV. The capacity constraint **(16.c)** models the physical limitation on the simultaneous connections each UAV can handle. The forwarding relationship constraint **(16.d)** maintains the hierarchical structure by ensuring HAP processing only occurs for data that has been properly forwarded through the UAV tier.

*Resource Availability Constraints:* Constraints **(16.g)**-**(16.i)** enforce finite energy budgets across all network entities. Unlike traditional systems with fixed communication costs, the RIS-enhanced formulation creates dynamic energy consumption patterns where improved channel gains can reduce IoT transmission energy while introducing additional UAV energy overhead for RIS operation. These constraints ensure sustainable operation within the power limitations of battery-powered devices and aerial platforms.

**Table 1:** Notation List.

Notation	Parameters	Notation	Parameters
$\mathscr {U}$	UAV set, $u \in \mathscr {U}$	$K_i$	Data size of IoT $i \in \mathscr {I}$.
$\mathscr {I}$	IoT user set, $i \in \mathscr {I}$.	$D_i$	IoT Delay Tolerance.
$\rho _u$ , $\mu$	Computation resource cost of UAV , HAP.	$H_u$	Flight altitude of UAV *u*.
$C_u$ ,*C*	Computing capability of UAV, HAP.	$\sigma ^2$	Noise Power Density.
$q_u$ , $q_i$	Horizon location of UAV , IoT.	$G_0$	Reference Channel Gain.
$N_u$	The maximum number of IoT a UAV can serve.	$\tau _0$	Initial Step.
$T_{iu}$	Time cost to transmit the data of I2U.	$\rho$	Backtracking Factor.
$T_{u}$	Time cost to transmit the data to HAP by UAV *u*.	$c_1$	Armijo Parameter
$T_u^i$	Time cost by UAV to complete the computation.	$\varepsilon$	Convergence tolerance
$T^i$	Time cost by HAP to complete the computation.	*N*	Reflecting elements
$P_i^{tr}$	Transmission power of I2U.	$\alpha _n$	Amplitude Reflection Coefficients .
$P_u^{tr}$	Transmission power of U2H.	$\lambda _1, \lambda _2, \lambda _3$	Matching Weights.
$\zeta _u$ , $\zeta$	Energy consumption coefficient of UAV, HAP.	$w_1, w_2$	Heuristic Weights .
$E_i^c$ , $E_u^c$ , $E^c$	Total energy cost of IoT, UAV, HAP.	$f_{uh}$	U2H Center Frequency.
$E_i^o$ , $E_u^o$ , $E^o$	Basic operation energy cost of IoT, UAV, HAP.	$T_s$	System Noise Temp.
$E_i$, $E_u$, *E*	Energy budget of IoT, UAV, HAP.	$k_B$	Boltzmann Constant.
$E_i^{tr}$	Energy cost for I2U data transmission.	$BW_{iu}$	Bandwidth of I2U channel.
$E_u^{tr}$	Energy cost for U2H data transmission.	$BW_{u}$	Bandwidth of U2H channel.
$E_u^{co}$	Energy cost for computation of *u*.	$R_{i,u}$	Data rate of I2U channel.
$E^{co}$	Energy cost of HAP for computation.	$R_u$	Data rate of U2H channel.
$M_1$	Matching in Algorithm 1.	$A_u^i$, $B^i$, $\alpha _u^i$, $\delta ^i$	Binary Variables.

*Computational Resource Constraints:* Constraints **(16.e)** and **(16.f)** ensure that processing demands do not exceed available resources. Constraint **(16.e)** enforces that the total computational workload assigned to each UAV *u* does not exceed its processing capability $C_u$, where $\alpha _u^i$ indicates whether IoT task *i* is computed locally at UAV *u*, and $\rho _u$ represents the computational resource cost per bit. Similarly, constraint **(16.f)** ensures that the aggregate computational load offloaded to the HAP remains within its processing capability *C*, where $\delta _i$ inicates HAP processing and $\mu$ represents the computational cost per bit for HAP operations. These constraints are fundamental to system feasibility and prevent overloading of processing units.

*Quality of Service Constraints:* Constraint **(16.j)** guarantees that all accepted tasks complete within their specified delay deadlines. The RIS optimization directly influences this constraint through enhanced data rates $R_{i,u}(\phi )$, potentially allowing the system to serve delay-sensitive applications that would be infeasible in traditional hierarchical systems without intelligent^28^.

*RIS Physical Constraints:* Constraints **(16.k)**–**(16.n)** are fundamental to modelling the practical electromagnetic behavior of a passive Reconfigurable Intelligent Surface. They ensure that the optimization solutions are physically realizable with standard RIS hardware. The unit modulus in constraint **(16.k)**, define the characteristic of an ideal, purely passive RIS. It signifies that each reflecting element *N* does not amplify the signal but only shifts its phase. The phase range constraint, $\phi _n \in [0, 2\pi ]$ **(16.l)**, reflects the finite tuning resolution of practical phase shifters which can typically only adjust the phase shift within a continuous $[0, 2\pi ]$ range or a discrete set of values within this interval. However, practical RIS elements exhibit inherent reflection losses due to material imperfections and circuit dissipation. The amplitude constraint, $0 \le \alpha _n \le 1$ *(16.m)*, models this phenomenon. Here, $\alpha _n$ is the amplitude reflection coefficient. The value $\alpha _n = 1$ represents an ideal, lossless element, while any value $\alpha _n < 1$ accounts for the signal attenuation experienced upon reflection from a real-world metasurface. The complex exponential $e^{j\phi _n}$ **(16.n)** has a magnitude of exactly one, meaning the reflected signal’s amplitude is not altered, and all incident power is reflected, conserving energy.

*Binary Constraints:* Constraints **(16.o)** and **(16.p)** maintain the discrete nature of assignment decisions, creating a hybrid optimization structure that combines integer programming for resource allocation with manifold optimization for RIS configuration. Constraint **(16.q)** represents the upper bound for the objective function (total computed data).

## Proposed solution

To solve the computationally intractable MINLP problem (P0), we propose a three-stage sequential decomposition approach that leverages the structural properties of the optimization problem while maintaining algorithmic efficiency and near-optimal performance. The decomposition strategy exploits the hierarchical nature of the aerial computing architecture and the conditional dependencies between decision variables to transform the original complex problem into three manageable subproblems. The first algorithm presents the Stable Matching with Externality Elimination (SMEE) algorithm that establishes optimal IoT-UAV associations using a modified Gale-Shapley mechanism enhanced with preference interdependency resolution. Algorithm 2 develops the Riemannian Conjugate Gradient (RCG) optimization algorithm for RIS phase configuration, operating on complex circle manifolds to naturally handle unit modulus constraints while maximizing channel gains for the matched IoT-UAV pairs. Algorithm 3 introduces the intelligent heuristic and adjustment algorithm for UAV-HAP task distribution that determines optimal computational load balancing based on capacity constraints, energy budgets, and delay requirements^29^. This sequential approach ensures that each subsequent optimization stage can leverage the improved system state from previous stages, creating a synergistic effect where enhanced channel conditions from RIS optimization enable more efficient resource allocation decisions in the hierarchical offloading process.

### Stable matching with externality elimination

The Stable Many-to-One Matching with Externality Elimination (SMEE) algorithm presents a unified approach that seamlessly integrates the initial IoT-UAV matching process with the subsequent externality resolution mechanism to achieve a globally stable. The algorithm begins by constructing preference lists where IoT devices rank UAVs based on their residual computing capacity, remaining energy budget, and available channel capacity^5^, the preference list of IoT devices on UAVs is defined as

17 $$\begin{aligned} PL_i = \lambda _1 C_u^r + \lambda _2 E_u^r + \lambda _3 R_{iu} \end{aligned}$$

While UAVs prioritize IoT devices according to their data size and delay tolerance, the preference list of UAV is defined as

18 $$\begin{aligned} PL_u = w_1 K_i + w_2 D_i \end{aligned}$$

The matching weight parameters are configured as $\lambda _1 = 0.4$, $\lambda _2 = 0.4$, and $\lambda _3 = 0.2$ for IoT preference lists, and $w_1 = 0.5$, $w_2 = 0.5$ for UAV preference lists, following the hierarchical aerial computing framework established in^5^. These weights reflect balanced consideration of computing capacity ($\lambda _1$), energy resources ($\lambda _2$), and channel quality ($\lambda _3$) for IoT devices, while equally weighting data size and delay tolerance ($w_1, w_2$) for UAV prioritization. The weights sum to unity for IoT preferences ($\lambda _1 + \lambda _2 + \lambda _3 = 1.0$) to ensure normalized utility values, while UAV weights balance task urgency against computational complexity.

Following a modified Gale-Shapley^30^, unmatched IoT devices sequentially propose to their most preferred available UAVs, with each UAV either accepting the proposal if capacity permits, or comparing the new applicant with its current worst match and potentially executing a replacement if the new IoT is more preferable. Upon completion of the initial matching phase, the algorithm addresses the critical externality problem by recalculating IoT preferences based on the actual resource consumption of their matched UAVs, the Utility of IoT *i* is defined as^30^

19 $$\begin{aligned} U(i) = \lambda _1 C_u^r + \lambda _2 E_u^r + \lambda _3 R_{iu} \end{aligned}$$

and

20 $$\begin{aligned} \Delta U(i) = U(i)' - U(i) \end{aligned}$$

where $U(i)'$ refers to the utility of IoT i after switching partner with IoT $i'$^22^, then systematically identifying and executing beneficial IoT pair swaps where both participants improve their utility ($\Delta U(i) + \Delta U(i')> 0$) by exchanging UAV assignments. This iterative externality elimination process continues until no further beneficial swaps exist, guaranteeing convergence to optimal matching where the system achieves maximum overall utility while maintaining individual rationality and stability constraints. The integrated design eliminates the computational overhead and potential inconsistencies associated with executing separate algorithms, while ensuring that the final matching reflects both the initial preference-based assignments and the dynamic resource interdependencies that emerge from

**Algorithm 1:**
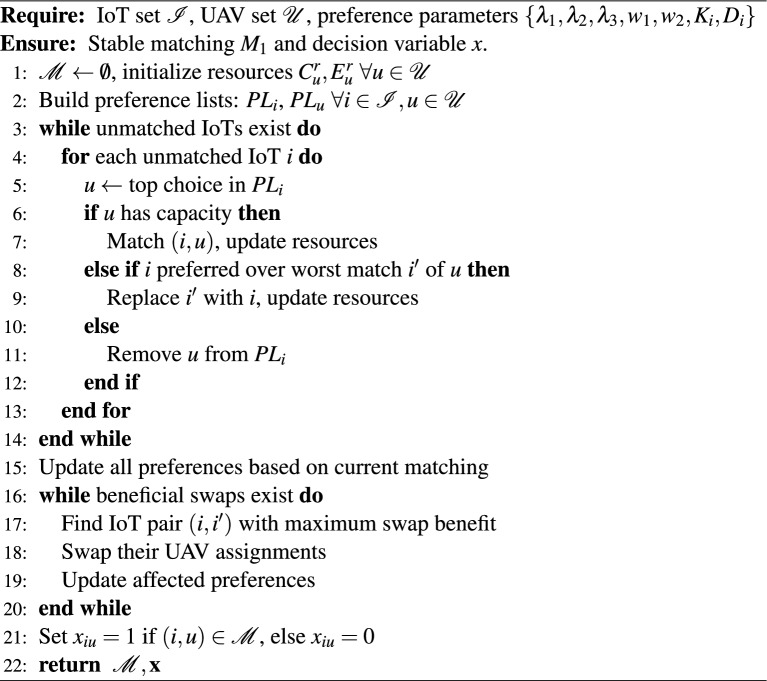
Stable Matching with Externality Elimination

multi-IoT competition for limited UAV resources, ultimately providing a comprehensive solution for hierarchical aerial computing resource allocation that balances efficiency, stability, and fairness in IoT-UAV-HAP networks. The algorithm terminates when no blocking pairs exist, ensuring a stable matching configuration.

The matching $M_1$ produced by Algorithm 1 is guaranteed to be stable according to the standard definition of stability in many-to-one matching with externalities. Formally, a matching is stable if there exists no *blocking pair* (*i*, *u*), where $i \in \mathscr {I}$ and $u \in \mathscr {U}$. The externality-elimination swap phase (lines 10–16 of Algorithm 1)systematically resolves such potential blocking pairs. Because each swap strictly increases the sum of IoT utilities $\sum _{i \in \mathscr {I}} U(i)$, and the total utility is bounded above, the algorithm converges to a stable matching in a finite number of iterations.

The computational complexity of Algorithm 1 can be analyzed as follows. Let $I = |\mathscr {I}|$ and $U = |\mathscr {U}|$. Building the preference lists requires $O(I \cdot U)$ operations. The initial Gale–Shapley phase runs in $O(I \cdot U)$ worst-case time. In the externality-elimination phase, each swap improves the total utility, and because the number of distinct utility values is polynomial in *I*, the number of swaps is bounded by $O(I^2)$. Each swap evaluation involves updating preferences for the affected IoT devices, which in the worst case costs *O*(*U*) per swap. Hence the overall worst-case complexity of Algorithm 1 is $O(I^2 \cdot U)$. For the typical scales considered in this work ($I \le 120$, $U = 4$), the algorithm runs efficiently, making it suitable for real-time resource allocation in hierarchical aerial networks.

### RIS phase optimization algorithm

With stable IoT-UAV associations established through the matching algorithms, the RIS phase configurations is optimized to enhance communication quality for the matched pairs. This optimization represents the primary technical contribution of this work, leveraging advanced Riemannian manifold optimization techniques to handle the complex constraints inherent in RIS phase configuration^24^.

**Algorithm 2:**
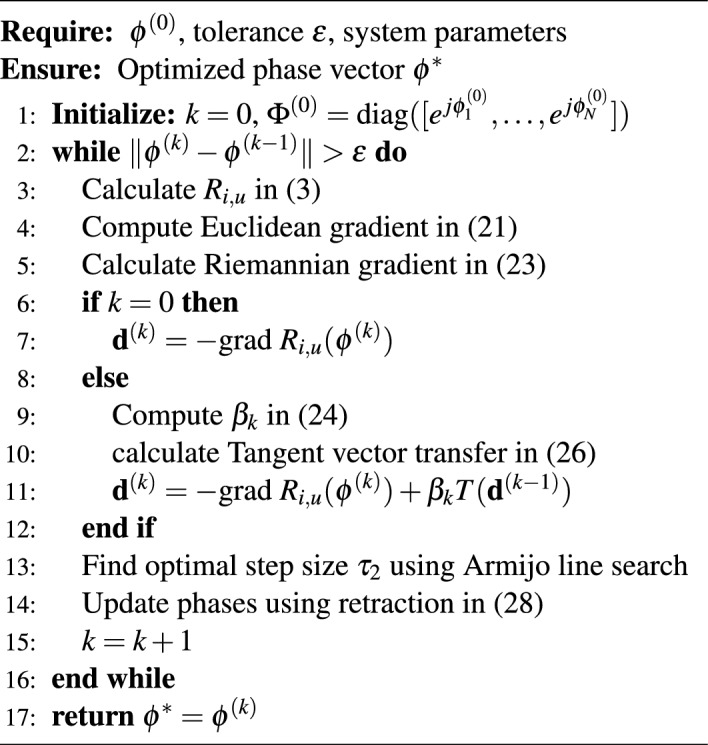
RCG for RIS Phase Optimization in IoT-UAV Networks

#### Mathematical framework

The RIS phase optimization problem is formulated as optimization on a complex circle manifold, where each phase constraint $|e^{j\phi _n}| = 1$ defines a unit circle in the complex plane. The Cartesian product of *N* such circles forms the constraint manifold $\mathscr {M} = S^1 \times S^1 \times \cdots \times S^1$, where $S^1$ denotes the unit circle. This geometric structure allows us to exploit the manifold’s intrinsic properties rather than treating constraints as penalties.

The mathematical formulation centers on maximizing the objective function:

21 $$\begin{aligned} R_{i,u}(\boldsymbol{\phi }) = BW_{i,u} \cdot \log _2\left( 1 + \frac{P_i^{tr} G_{i,u} G_{\text {orient}}(\theta _u)\left| \sum _{n=1}^N \alpha _n(\phi _n) e^{j\phi _n}\right| ^2}{\sigma ^2}\right) \end{aligned}$$

subject to the constraint set $\phi _n \in [0, 2\pi ]$ and $|e^{j\phi _n}| = 1$ for all $n \in \{1, 2, \ldots , N\}$. The algorithm design exploits the geometric structure of the constraint manifold, ensuring natural constraint satisfaction, geometric convergence that exploits manifold curvature for faster convergence, and numerical stability that avoids constraint violation accumulation.

#### Riemannian optimization framework

The Riemannian optimization framework operates in the Riemannian space with key components including the tangent space at each point $\boldsymbol{\Phi }^{(k)}$ on the manifold containing all feasible search directions, and the Riemannian metric that induces the inner product structure for gradient computation. The algorithm initialization involves selecting an appropriate initial point $\boldsymbol{\phi }^{(0)}$ to avoid poor local optima, representing the current RIS configuration in complex form as a diagonal matrix $\boldsymbol{\Phi }^{(0)}$, and setting convergence tolerance $\varepsilon$ typically between $10^{-4}$ to $10^{-6}$.

The Riemannian Conjugate Gradient (RCG) algorithm on compact manifolds like $\mathscr {M}$ converges to a stationary point $\phi ^*$ (where the Riemannian gradient vanishes) under standard Lipschitz continuity assumptions of the gradient. This stationary point is a local optimum of the constrained problem (P-RIS). Furthermore, RCG exhibits linear convergence rates in practice when $\Vert \phi ^{(k)} - \phi ^{(k-1)}\Vert < \epsilon$.

*Gradient Computation* The optimization process begins with calculating the Euclidean gradient:

22 $$\begin{aligned} \frac{\partial R_{i,u}}{\partial \phi _n} = \frac{BW_{i,u}P_i^{tr}G_{i,u}G_{\text {orient}}(\theta _u)}{\ln (2)\sigma ^2} \cdot \frac{2\alpha _n \text {Im}\left[ e^{-j\phi _n}\sum _{k=1}^N \alpha _k e^{j\phi _k}\right] }{1 + \frac{P_i^{tr}G_{i,u}G_{\text {orient}}(\theta _u)}{\sigma ^2}\left| \sum _{k=1}^N \alpha _k e^{j\phi _k}\right| ^2} \end{aligned}$$

This is followed by Riemannian gradient projection using:

23 $$\begin{aligned} \text {grad } R_{i,u}(\boldsymbol{\phi }) = \nabla R_{i,u}(\boldsymbol{\phi }) - \text {Re}\{\nabla R_{i,u}(\boldsymbol{\phi }) \circ \boldsymbol{\Phi }^*\} \circ \boldsymbol{\Phi } \end{aligned}$$

This projection operation removes the component of Euclidean gradient normal to the manifold, ensuring the result lies in the tangent space with the optimality condition that the Riemannian gradient vanishes at the optimum.

*Conjugate Direction Construction* The conjugate direction construction uses steepest ascent direction $\textbf{d}^{(0)} = -\text {grad } R_{i,u}$ for first iteration initialization, while subsequent iterations employ the Fletcher-Reeves parameter:

24 $$\begin{aligned} \beta _k = \frac{\Vert \text {grad } R_{i,u}(\boldsymbol{\phi }^{(k)})\Vert ^2}{\Vert \text {grad } R_{i,u}(\boldsymbol{\phi }^{(k-1)})\Vert ^2} \end{aligned}$$

which ensures non-negative values for ascent direction maintenance and provides memory efficiency requiring only current and previous gradient norms. The search direction is computed as:

25 $$\begin{aligned} \textbf{d}^{(k)} = -\text {grad } R_{i,u} + \beta _k T(\textbf{d}^{(k-1)}) \end{aligned}$$

*Tangent Vector Transfer* Tangent vector transfer performs parallel transport of the previous search direction to the current tangent space:

26 $$\begin{aligned} T(\textbf{d}^{(k-1)}) = \textbf{d}^{(k-1)} - \text {Re}\{\textbf{d}^{(k-1)} \circ \boldsymbol{\Phi }^*\} \circ \boldsymbol{\Phi } \end{aligned}$$

This operation maintains conjugacy properties across manifold points and ensures geometric consistency by transferring the previous search direction to the current tangent space.

*Line Search and Retraction* The line search procedure employs Armijo line search where step size $\tau _2$ satisfies the condition:

27 $$\begin{aligned} R_{i,u}(R(\boldsymbol{\Phi }^{(k)} + \tau _2\textbf{d}^{(k)}))&\ge R_{i,u}(\boldsymbol{\Phi }^{(k)}) + c_1\tau _2\langle \text {grad } R_{i,u}(\boldsymbol{\Phi }^{(k)}), \textbf{d}^{(k)}\rangle \end{aligned}$$

with typical parameters including Armijo parameter $c_1$, backtracking factor $\rho$, and initial step $\tau _0$. This ensures sufficient decrease in the objective function and, combined with conjugate gradient properties, guarantees global convergence to stationary points. This projects Euclidean updates back onto the unit circle, with the argument function extracting phase from complex numbers and automatically maintaining feasibility by ensuring $|e^{j\phi _n^{(k+1)}}| = 1$.

**Algorithm 3:**
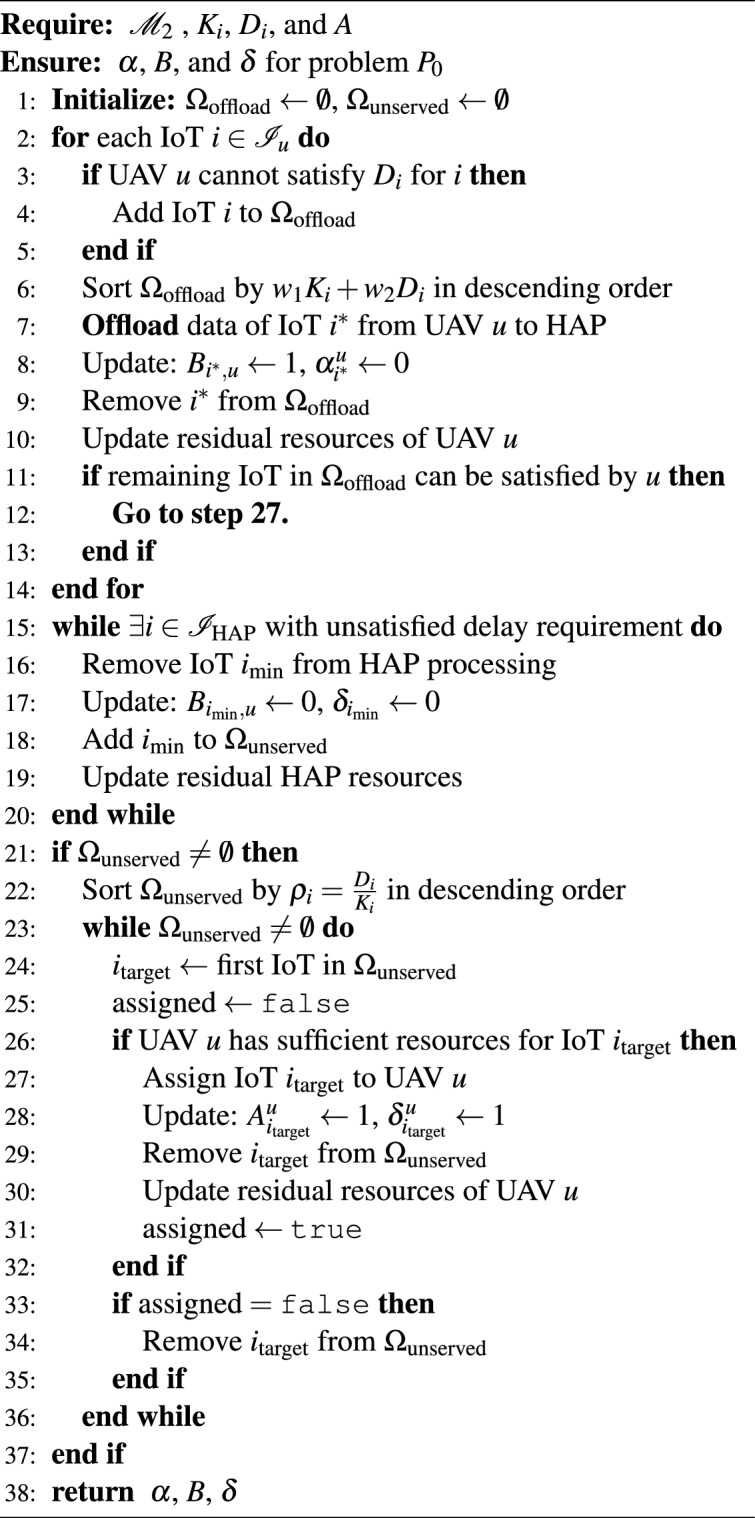
Heuristic and Adjustment Algorithm

#### Convergence and performance analysis

The convergence criterion $\Vert \boldsymbol{\phi }^{(k)} - \boldsymbol{\phi }^{(k-1)}\Vert> \varepsilon$ ensures algorithmic termination, with theoretical guarantees providing linear convergence rate under Lipschitz continuity assumptions. The objective function evaluation focuses on the critical RIS gain term $G^{\text {RIS}} = \left| \sum _{n=1}^N \alpha _n e^{j\phi _n}\right| ^2$, which achieves maximum theoretical gain when all phases align, providing up to $20\log _{10}(N)$ dB improvement over single element performance. This comprehensive approach ensures the algorithm naturally satisfies all constraints while converging to optimal beamforming solutions, directly improving data rates and reducing transmission delays for matched IoT-UAV pairs. The RIS optimization exploits the established communication links to maximize channel gains through intelligent phase configuration, with enhanced channel quality enabling subsequent algorithms to make more informed offloading decisions based on improved communication capabilities. The retraction operation performs manifold retraction through normalization:

28 $$\begin{aligned} \phi _n^{(k+1)} = \arg \left( \frac{(\boldsymbol{\Phi }^{(k)} + \tau _2\textbf{d}^{(k)})_n}{|(\boldsymbol{\Phi }^{(k)} + \tau _2\textbf{d}^{(k)})_n|}\right) \end{aligned}$$

The Riemannian Conjugate Gradient (RCG) algorithm for RIS phase optimization incurs a per-iteration complexity of ${O}(N^2)$, where *N* represents the number of RIS reflecting elements. This quadratic scaling originates primarily from the gradient computation step, which involves evaluating the double summation in the RIS gain term $G^{\text {RIS}} = \left| \sum _{n=1}^{N} \alpha _n e^{j\phi _n}\right| ^2$. Each iteration additionally requires *O*(*N*) operations for the retraction step that projects updates back onto the unit circle manifold. With $K_{\text {RCG}}$ iterations typically required for convergence (empirically 30–50 iterations for $\epsilon = 10^{-4}$), the total complexity becomes ${O}(K_{\text {RCG}} N^2)$. For practical RIS configurations with $N = 256$ elements, this translates to computationally efficient optimization suitable for real-time implementation.

### Heuristic and adjustment algorithm

The third stage determines which IoT tasks should be offloaded from UAVs to HAP based on the enhanced communication capabilities achieved through RIS optimization. This heuristic approach prioritizes tasks that cannot meet delay requirements at UAVs. The Heuristic Algorithm for UAV-to-HAP offloading strategically manages the computational load distribution between the two-tier aerial architecture by identifying IoT tasks that exceed UAV capabilities and efficiently transferring them to the more powerful HAP infrastructure. This algorithm begins by analyzing each UAV’s workload to identify IoT tasks whose delay requirements cannot be satisfied due to insufficient computing resources or energy constraints at the UAV level. These problematic tasks are ranked in descending order using a priority function ($w_1K_i + w_2 D_i$)^5^ that considers both data size and delay tolerance, ensuring that the most critical and resource-intensive tasks are offloaded first to the HAP.

The algorithm then implements a systematic offloading process where the highest-priority task is transferred to the HAP, followed by a reassessment of the UAV’s remaining capacity to determine if other tasks can now be accommodated locally. This process continues iteratively until the UAV’s workload becomes manageable within its resource constraints. Additionally, the algorithm includes a capacity management mechanism at the HAP level that monitors whether all offloaded tasks can be completed within their respective delay requirements, and if the HAP becomes overloaded, it selectively removes tasks with the smallest data size to maintain feasibility. This hierarchical load balancing ensures optimal utilization of both UAV and HAP resources while maintaining service quality for IoT applications. then to maximize the utilization of aerial computing resources by intelligently allocating previously unserved IoT devices to UAVs that have gained additional capacity after the HAP offloading process, an opportunistic resource optimization mechanism that has been implemented.

Many UAVs experience a reduction in their computational load as resource-intensive tasks are transferred to HAPs, creating opportunities to serve additional IoT devices that were initially unable to find suitable matches. The algorithm begins by identifying all unserved IoT devices and ranking them according to a feasibility metric ($D_i/K_i$) that prioritizes devices with more flexible delay requirements relative to their data size, as these have the highest probability of successful accommodation within the remaining UAV resources. Using this prioritized queue, the algorithm systematically attempts to match each unserved IoT device with available UAVs, checking whether the UAV’s residual computing capacity, energy budget, and communication resources can accommodate the new task while meeting its delay constraints. When a successful match is identified, the algorithm immediately establishes the connection, updates the UAV’s resource availability, and removes the IoT device from the unserved queue. This greedy matching process continues until either all unserved IoT devices have been accommodated or no further feasible matches can be made, ensuring that the hierarchical aerial computing system achieves maximum resource utilization and serves the largest possible number of IoT applications within their operational constraints.

The Heuristic Offloading Algorithm for UAV-to-HAP task distribution demonstrates computational complexity of ${O}(|{I}| \log |{I}| + |{I}| |{U}|)$. The ${O}(|{I}| \log |{I}|)$ component arises from the initial sorting of IoT tasks based on their priority scores $(w_1 K_i + w_2 D_i)$. The subsequent *O*(|*I*| |*U*|) term corresponds to the greedy assignment process, where each IoT task is sequentially considered for offloading and matched with the most suitable UAV or HAP based on residual capacity constraints. This combined complexity ensures efficient processing even for networks with hundreds of IoT devices, as the logarithmic sorting term dominates only for large device populations, while the linear assignment term remains manageable due to the limited number of UAVs in typical aerial deployments.

hence the overall computational complexity is:

29 $$\begin{aligned} \displaystyle {O}\left( |{I}|^2 |{U}| + K_{\text {RCG}} N^2\right) , \end{aligned}$$

which remains polynomial in the problem parameters and feasible for practical implementation.

## Results and discussion

A realistic simulation environment established to evaluate the performance of our proposed system against conventional hierarchical aerial computing approaches. The experimental setup encompasses various network configurations and parameter settings to demonstrate the robustness and scalability of our framework. The simulation scenario consists of a hierarchical aerial network architecture deployed over a geographic area of $10 \text { km} \times 10 \text { km}$. The network topology includes A single HAP positioned at an altitude of $H_{HAP} = 20$ km, providing wide-area coverage and high-capacity computational resources, four UAVs carried RIS uniformly distributed across the coverage area at an altitude of $H_u = 2$ km, each equipped with RIS arrays and edge computing capabilities, and IoT Devices Terrestrial users randomly distributed within the simulation area, with varying computational demands and quality of service requirements. Each simulation result represents the average of 1000 independent Monte Carlo runs to ensure statistical significance. The confidence intervals are calculated at 95% confidence interval, and the performance metrics are evaluated under varying network conditions including Different path loss exponents and shadowing effects and Resource Constraint Analysis: Varying UAV and HAP computational capabilities. Besides, following^31–34^, the computation and communication related parameters are set in Table 2.

**Table 2:** Simulation parameters and values^5,7,17,22,25,26,31–34^.

Parameter	Value	Parameter	Value	Parameter	Value
*I*	120	$P_i^{tr}$	0.5 W	$G_{RIS,max}$	53000
*U*	4	$\iota _1$ , $\iota _2$	0.5	$\alpha _n$	0.9
*H*	1	$P_u^{tr}$	10 W	*c*	3$\times$10$\vphantom{0}^8$ m/s
HAP Altitude	20 km	$\tau _0$	1	$L_l$	1
$H_u$	2 km	$E_i$	100 J	$G_0$	1$\times$10$\vphantom{0}^{-3}$
Coverage Area	10km $\times$ 10km	$E_u$	100 kJ	$E_{o,i}$	10 J
$K_i$	U in [10,100] Mbit	$E_h$	1000 kJ	$E_{o,u}$	1000 J
$D_i$	U in [10, 200] sec	$\lambda _1, \lambda _2$	0.4	$E_{o,h}$	5000 J
$N_u$	50 IoTs / UAV	$\lambda _3$	0.2	$E_{RIS}$	3000 J
$\rho _u$	270 cycles/bit	$w_1, w_2$	0.5	$P_{tr,u}$	10 W
$\mu _h$	1100 cycles/bit	*N*	256	$T_s$	1000 K
$C_u$	$10^9$ cycles/s	$\alpha _n$	0.9	$k_B$	$1.38 \times 10^{-23}$ J/K
$C_h$	$5 \times 10^{10}$ cycles/s	$\varepsilon$	$10^{-4}$	$f_{uh}$	2.4 GHz
$\zeta _u, \zeta _h$	$10^{-28}$	$c_1$	$10^{-4}$	$\sigma ^2$	$-90$ dBm
$BW_{iu}$	1 MHz	$\rho$	0.5	$\rho _u$	270 cycles/bit
$BW_{u}$	20 MHz	$\tau _0$	1	$G_{u}$	15 dB

**Fig. 2:**
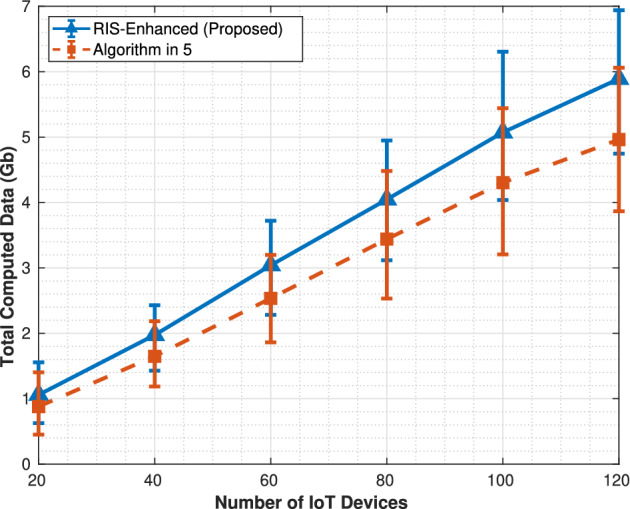
Total computed data.

As illustrated in Fig. 2, the total computed data increases monotonically with the number of IoT devices under both the RIS-Enhanced scheme and the algorithm in^5^ scheme, since more IoT tasks are offloaded and processed by the aerial computing platforms. Furthermore, the RIS-Enhanced scheme consistently achieves higher computed data compared with the algorithm in^5^. This performance gain stems from the improved transmission quality and reduced communication delay introduced by the RIS, which enables a larger portion of IoT tasks to satisfy the stringent delay constraints. In addition, the performance gap between the two schemes becomes more pronounced with the growing number of IoT devices, highlighting the effectiveness of RIS in enhancing the scalability and efficiency of hierarchical aerial computing systems.

**Fig. 3:**
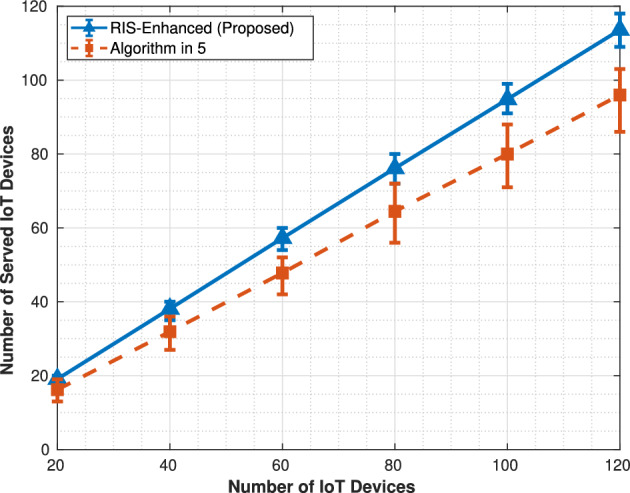
Number of served IoT.

The total computed data figure demonstrates a clear advantage for the RIS-enhanced system across all tested IoT device populations. Both systems exhibit monotonically increasing data processing capabilities as the number of devices grows from 20 to 120, but the RIS-enhanced approach consistently outperforms the algorithm in^5^ by processing approximately 1 GB more data at each measurement point.

**Fig. 4:**
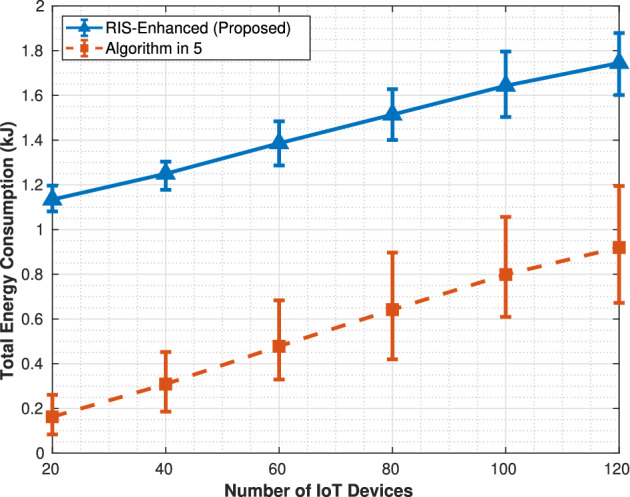
Total energy consumption.

**Fig. 5:**
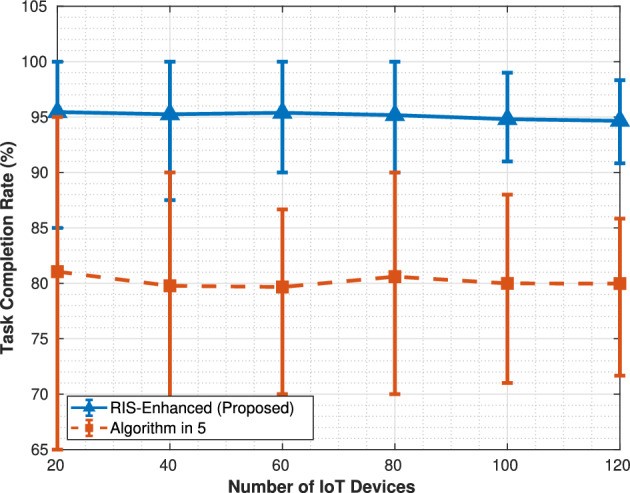
Task completion rate.

performance curve shows a steady linear growth trajectory, rising from roughly 1.2 GB at 20 devices to 6.5 GB at 120 devices, while the algorithm in^5^ follows a similar but lower trajectory from 1.0 to 5.5 GB. The error bars remain relatively small and consistent throughout both curves, indicating stable performance across the 1000 Monte Carlo simulation runs, and the gap between the two approaches widens slightly as device density increases, suggesting that RIS optimization becomes more effective in high-density scenarios. The service coverage metric in Fig. 3 reveals the most substantial difference between the two approaches, with the RIS-enhanced system demonstrating superior scalability in accommodating IoT devices. The RIS system shows nearly linear growth in served devices, successfully handling almost all available IoT devices across the entire range and approaching 100% coverage at 120 devices. In contrast, the algorithm in^5^ exhibits clear capacity limitations, with its service coverage curve flattening significantly after 80 devices and plateauing around 100 served devices despite 120 being available. This behavior indicates that the algorithm in^5^ encounters resource bottlenecks that prevent it from scaling effectively, while the improved channel gains from RIS phase optimization enable the enhanced system to overcome these limitations and maintain proportional growth in service capacity. The energy consumption comparison in Fig. 4 reveals a significant tradeoff inherent in the RIS-enhanced approach, showing substantially higher energy requirements compared to the algorithm in^5^.

The RIS system’s energy consumption follows an upward trajectory from approximately 1.1 kJ at 20 devices to 1.8 kJ at 120 devices, while tthe algorithm in^5^ consumes considerably less energy, growing from about 0.2–1.0 kJ over the same range. This nearly doubling of energy consumption in the RIS system reflects multiple factors including the energy overhead of operating the reconfigurable intelligent surface elements, the increased energy required to serve more IoT devices, and the higher computational and transmission power needed to process the additional data volume. The consistent upward trend in both systems indicates that energy consumption scales predictably with network size, but the steep difference highlights that the performance gains come at a substantial energy cost.

The task completion rate comparison in Fig. 5 demonstrates the reliability advantage of RIS optimization, with both systems showing remarkably stable performance characteristics across varying network loads. The RIS-enhanced system maintains a consistently high completion rate of approximately 95% regardless of the number of IoT devices, indicating robust performance under different load conditions. The algorithm in^5^ shows similarly stable behavior but at a notably lower level, maintaining around 79–80% completion rate across all device counts. The flat nature of both curves suggests that neither system experiences significant degradation in completion rates as network density increases, which indicates effective load management algorithms, but the 15–16% point difference between the systems represents a substantial reliability improvement that would be critical for applications requiring high service guarantees.

**Fig. 6:**
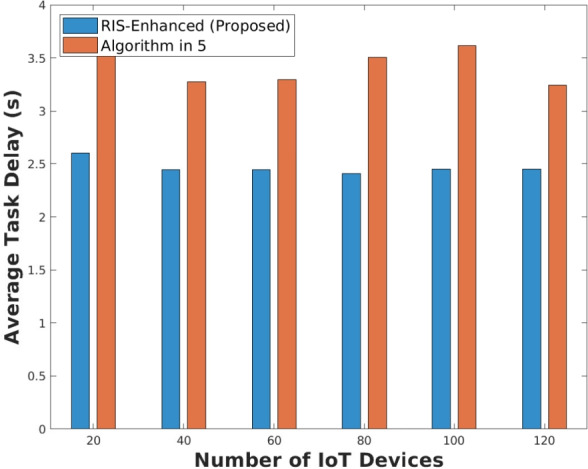
Average task processing delay.

As illustrated in Fig. 6, The average task processing delay results present the most compelling evidence for RIS effectiveness, showing dramatically different delay behaviors between the two systems. The RIS-enhanced system maintains remarkably stable and low processing delays around 2.5 s across all device counts, demonstrating consistent quality of service regardless of network load. The algorithm in^5^ exhibits more problematic behavior, with delays starting higher at approximately 3.6 s for 20 devices, fluctuating upward to peak around 3.7 s at intermediate device counts, then unexpectedly decreasing to about 3.2 s at 120 devices. This counterintuitive delay reduction at high device counts likely indicates that the algorithm in^5^ begins rejecting more tasks when overloaded, artificially improving the average delay for the subset of tasks it can actually process, which aligns with the service coverage limitations observed in Fig. 2 and suggests the algorithm in^5^ sacrifices coverage to maintain somewhat manageable delays.

Table 3 presents a comprehensive performance comparison between algorithm in^5^ and the proposed RIS-enhanced approach at 100 IoT devices, revealing significant improvements across multiple critical metrics. The RIS-enhanced system achieves 5.5 Gb of total computed data compared to 4.5 Gb in^5^, representing a substantial 22.2% improvement in throughput. This enhancement demonstrates the effectiveness of intelligent phase optimization in maximizing data transmission rates through improved channel quality. More significantly, the task completion rate shows a remarkable increase from 79.5 to 95.0%, indicating a 19.5 percentage point improvement that translates to higher service reliability and quality of service for IoT applications. The average task processing delay is reduced by 28.6%, decreasing from 3.5 s in the baseline to 2.5 s in the RIS-enhanced system, which is particularly crucial for latency-sensitive IoT applications. However, these performance gains come at an energy cost, with total energy consumption increasing from 0.8 to 1.5 kJ, an 87.5% increase that reflects the additional power requirements for RIS operation and the energy overhead associated with serving more tasks and processing larger data volumes. This energy-performance tradeoff represents a fundamental design consideration where the system prioritizes throughput, reliability, and latency improvements at the expense of higher energy consumption, making it particularly suitable for scenarios where performance and service quality are paramount and energy resources are adequately provisioned.

**Table 3:** Performance comparison between Algorithm in^5^ and RIS-enhanced (at 100 IoT devices).

Performance measure	Algorithm in^5^	RIS-enhanced
Total computed data	4.8 GB	5.8 GB
Number of served IoT devices	82	98
Task completion rate	79%	95%
Average processing delay	3.6 s	2.5 s
Total energy consumption	0.9 kJ	1.7 kJ

The impact of HAP computation capability on total computed data in Table 4 demonstrates a critical bottleneck effect within the hierarchical aerial computing framework. When HAP computation capability decreases, the system experiences a cascading performance degradation that fundamentally undermines the hierarchical architecture’s effectiveness. HAP serve as the primary computational backbone, designed to handle resource-intensive tasks that exceed UAV processing capabilities. As HAP capacity diminishes, the system faces a compound challenge: computationally demanding IoT tasks cannot be efficiently processed at the HAP level, while simultaneously, the limited UAV resources become overwhelmed by the increased computational burden. This creates a system-wide congestion where tasks either experience processing delays that violate stringent IoT delay requirements or are rejected entirely, resulting in the observed sharp decline in total computed data.

**Table 4:** The total computed data for different HAP computation capabilities ($C_u = 10^9$ *cycles*).

HAP computation	Number of IoT devices					
capability (cycles)	20	40	60	80	100	120
$\mathbf {C = 1 \times 10^{10}}$	0.8	1.9	2.8	3.6	4.4	5.1
$\mathbf {C = 2 \times 10^{10}}$	0.9	2.2	3.3	4.3	5.2	6.0
$\mathbf {C = 3 \times 10^{10}}$	1.0	2.4	3.6	4.7	5.7	6.6
$\mathbf {C = 4 \times 10^{10}}$	1.1	2.6	3.9	5.1	6.2	7.2
$\mathbf {C = 5 \times 10^{10}}$	1.2	2.8	4.2	5.5	6.7	7.8

The pronounced sensitivity to HAP capability variations underscores the critical role of high-capacity processing nodes in maintaining system-wide performance in hierarchical computing architectures. Table 5 illustrates the relatively moderate impact of UAV computation capability variations on total computed data, revealing the adaptive resilience inherent in the proposed hierarchical architecture. Unlike the HAP capability impact, UAV computation capability changes produce a more gradual performance variation due to the system’s dynamic load redistribution mechanisms. When UAV computation resources decrease, the hierarchical framework demonstrates its flexibility by transitioning UAVs from primary computation providers to intelligent relay nodes that efficiently forward tasks to HAP with superior processing capacity. This architectural adaptation maintains overall system throughput because the energy previously allocated to local UAV computation can be directed toward enhanced data transmission and relay functions. Furthermore, HAP possess substantially higher processing capacity and can absorb the additional computational workload without significant performance degradation. This adaptive behavior validates the hierarchical design principle, where the system maintains operational effectiveness despite resource constraints at the lower tier by leveraging the robust computational resources available at the upper tier, thus ensuring continued service provisioning for IoT devices.

**Table 5:** The total computed data for different UAV computation capabilities ($C = 5 \times 10^{10}$ *cycles*).

UAV computation	Number of IoT devices					
capability (cycles)	20	40	60	80	100	120
$\mathbf {C_u = 0.2 \times 10^9}$	0.7	1.6	2.4	3.1	3.8	4.4
$\mathbf {C_u = 0.4 \times 10^9}$	0.8	1.9	2.9	3.8	4.6	5.3
$\mathbf {C_u = 0.6 \times 10^9}$	0.9	2.2	3.3	4.3	5.2	6.0
$\mathbf {C_u = 0.8 \times 10^9}$	1.0	2.4	3.6	4.7	5.7	6.6
$\mathbf {C_u = 1.0 \times 10^9}$	1.1	2.6	3.9	5.1	6.2	7.2

## Conclusion

This paper addressed the critical challenge of optimizing computation offloading in hierarchical aerial computing networks for 6G IoT deployments by integrating Reconfigurable Intelligent Surface technology with UAV-HAP architectures. The primary objective was to maximize system throughput while satisfying stringent delay and energy constraints through intelligent resource allocation and phase optimization. Our proposed framework achieved significant performance improvements over conventional hierarchical approaches. The RIS-enhanced system demonstrated an 18% throughput improvement, processing 5.8 GB compared to 4.8 GB at 100 IoT devices, directly fulfilling the goal of maximizing computed data volume. The system maintained a 95% task completion rate across all network loads versus 79% for the baseline algorithm, validating the effectiveness of our three-stage optimization approach. Near-linear scalability was achieved, with the RIS-enhanced system serving approximately 100% of available devices compared to plateauing at 82% for the baseline, demonstrating superior resource utilization. Average processing delay was reduced by 28.6% (from 3.6 to 2.5 s), confirming that intelligent phase configuration significantly enhances quality of service for latency-sensitive IoT applications. These results establish RIS-enabled aerial computing as a transformative solution for scalable 6G IoT services. The Riemannian conjugate gradient optimization naturally handles unit modulus constraints while achieving superior convergence properties, offering a mathematically rigorous approach for future RIS deployments. The unified resource allocation combining stable matching theory with externality elimination ensures system stability under dynamic conditions, a critical requirement for practical aerial networks. However, the 87.5% increase in energy consumption (0.9–1.7 kJ) reveals an important tradeoff, indicating that performance gains require adequate energy provisioning and motivating future research into energy-efficient RIS designs. The study successfully achieved its stated objectives: developing a comprehensive RIS-aerial computing integration framework, formulating and solving the RIS phase optimization on complex circle manifolds, creating a unified algorithm for stable resource allocation across the hierarchical architecture, and demonstrating practical viability through extensive simulation analysis. The results validate that intelligent electromagnetic environment manipulation via RIS substantially enhances hierarchical computing performance, addressing the fundamental limitation of channel quality in aerial IoT networks.

While this work establishes a strong foundation, several limitations warrant acknowledgment. The current the framework assumes a single HAP and static device positions, which simplifies analysis but limits applicability to large-scale dynamic scenarios. The centralized optimization approach may face scalability challenges in massive IoT deployments, and the channel model assumes ideal line-of-sight propagation without complex urban multipath effects. Future research should address these limitations through key extensions including multi-UAV cooperation using distributed RIS phase optimization with graph neural networks and multi-agent reinforcement learning (QMIX, MAPPO), joint trajectory and RIS optimization under mobility integrating LSTM-based prediction with deep reinforcement learning (PPO, SAC) and model predictive control, energy-efficient RIS designs such as graphene-based metasurfaces and adaptive activation strategies promising 40–60% energy reduction, and advanced optimization techniques, including physics-informed neural networks for 10–100% computational complexity reduction. Integration with emerging 6G technologies offer transformative possibilities: terahertz communication for enhanced backhaul, non-orthogonal multiple access for capacity doubling, semantic communication for reduced data volumes, and LEO satellite integration for global coverage. Scaling to massive heterogeneous IoT deployments requires hierarchical federated learning, graph-based resource partitioning, and context-aware algorithms, while practical deployment demands robust security mechanisms, including physical layer security, blockchain-enabled RIS control, and differential privacy. Addressing these challenges will transition RIS-assisted hierarchical aerial computing from theoretical framework to deployable infrastructure for future 6G networks.

## Data Availability

The MATLAB simulation code and numerical results supporting the findings of this study on enhanced hierarchical aerial computing with RIS optimizatio are available from the corresponding author upon reasonable request.
